# Learning Curve for Robotic-Assisted Cholecystectomy

**DOI:** 10.1001/jamasurg.2024.1221

**Published:** 2024-05-22

**Authors:** Kyle H. Sheetz, Jyothi R. Thumma, Stanley Kalata, Edward C. Norton, Justin B. Dimick

**Affiliations:** 1 Department of Surgery, University of Michigan, Ann Arbor; 2 Center for Healthcare Outcomes and Policy, University of Michigan, Ann Arbor; 3 Department of Health Management and Policy, University of Michigan, Ann Arbor; 4 Department of Economics, University of Michigan, Ann Arbor; 5 Surgical Innovation Editor, *JAMA Surgery*

## Abstract

This cohort study evaluates the learning curve for robotic-assisted cholecystectomy among US surgeons from 2010 through 2019.

During the past decade, robotic-assisted cholecystectomy use has increased more than 30-fold across the United States.^1^ Surgeons gravitated toward this approach for numerous reasons, including promise that the new technology will make the operation safer and to refine their robotic surgical skills with a familiar operation. Recent evidence suggests bile duct injuries, an uncommon yet morbid technical complication, are more common with robotic-assisted than traditional laparoscopic cholecystectomy.^2^ Critics suggest that bile duct injury rates may only be higher for surgeons still progressing along their learning and therefore may not accurately reflect bile duct injury rates in real-world practice. We evaluated the learning curve for robotic-assisted cholecystectomy among a cohort of US surgeons from 2010 through 2019, when use of this approach moved out of specialty centers and was rapidly adopted into general practice across the country.

## Methods

We used 100% nationwide Medicare claims to identify 637 765 patients undergoing cholecystectomy from 2010 through 2019. We identified 4443 individual surgeons by unique National Provider Identifier in Part B claims. Outcomes, data definitions, coding for each approach, and analytic strategy have been previously described.^2^ We assumed that because robotic-assisted cholecystectomy was uncommon prior to 2010, surgeons’ first cases in the Medicare claims approximate their true first operation. Using logistic regression controlling for patient and hospital characteristics, we estimated bile duct injury rates for surgeons’ first to *n*th cases sequentially. Case volumes in the Medicare population were inflated to reflect true volumes by dividing hospitals’ total number of Medicare discharges by total admissions from the American Hospital Association survey. We used the adjusted national mean bile duct injury rate for laparoscopic cholecystectomy as a benchmark for the robotic-assisted learning curve since evidence suggests surgeons overcome their learning curve for this operation by the end of formal training.^3^ We also performed sensitivity analyses restricted to high-volume surgeons and using linear splines to account for different slopes across the learning curve (eAppendix in Supplement 1). Two-tailed *P* < .05 was considered statistically significant.

This study was deemed exempt by the institutional review board of the University of Michigan. This study used deidentified secondary data for which informed consent was not applicable.

## Results

Patient characteristics were similar between those undergoing laparoscopic vs robotic-assisted cholecystectomy (Table). While more patients undergoing laparoscopic cholecystectomy had acute cholecystitis (88.0% vs 77.9%; *P* < .001), the opposite was observed for symptomatic cholelithiasis (6.8% vs 11.6%; *P* < .001). Most surgeons (3867 [87.0%]) performed fewer than 10 robotic-assisted cholecystectomies (Figure, A). Bile duct injury rates following robotic-assisted cholecystectomy decreased with increasing experience (Figure, B). To reach equivalent bile duct injury rates with traditional laparoscopic cholecystectomy, surgeons would need to perform between 300 and 450 robotic-assisted cholecystectomies.

**Table.** Patient, Hospital, and Surgeon Characteristics From 2010 Through 2019

Characteristic	No. (%)		*P* value
	Laparoscopic cholecystectomy (n = 622 620)	Robotic-assisted cholecystectomy (n = 15 145)	
**Patients**			
Age, mean (SD), y	74.6 (10.1)	73.2 (10.3)	<.001
Sex			
Female	328 845 (52.8)	8202 (54.2)	.001
Male	293 775 (47.2)	6943 (45.8)	
Race and ethnicity^a^			
Asian	10 919 (1.8)	311 (2.1)	<.001
Black	45 172 (7.3)	1303 (8.6)	
Hispanic	18 168 (2.9)	475 (3.1)	
North American native	4719 (0.8)	86 (0.6)	
White	527 642 (84.7)	12 496 (82.5)	
Other race	10 400 (1.7)	285 (1.9)	
Unknown	5600 (0.9)	189 (1.2)	
Comorbidities			
Hypertension	459 848 (73.9)	11 426 (75.4)	<.001
Fluid and electrolyte disorders	195 675 (31.4)	4794 (31.7)	.55
Diabetes without chronic complications	139 827 (22.5)	3152 (20.8)	<.001
Obesity	99 368 (16.0)	2985 (19.7)	<.001
Chronic pulmonary disease	120 671 (19.4)	2942 (19.4)	.89
Hypothyroidism	105 187 (16.9)	2735 (18.1)	<.001
Deficiency anemia	97 117 (15.6)	2629 (17.4)	<.001
Kidney failure	94 193 (15.1)	2467 (16.3)	<.001
Congestive heart failure	82 078 (13.2)	1932 (12.8)	.13
Diabetes with chronic complications	53 393 (8.6)	1868 (12.3)	<.001
Depression	61 703 (9.9)	1687 (11.1)	<.001
Liver disease	44 666 (7.2)	1428 (9.4)	<.001
Neurological disorder	47 383 (7.6)	1144 (7.6)	.80
Valvular disease	45 720 (7.3)	1140 (7.5)	.39
Weight loss	36 649 (5.9)	1022 (6.7)	<.001
Peripheral vascular disease	42 004 (6.7)	956 (6.3)	.04
Coagulopathy	35 219 (5.7)	866 (5.7)	.75
Diagnosis			
Cholecystitis	547 801 (88.0)	11 791 (77.9)	<.001
Cholangitis	24 963 (4.0)	637 (4.2)	.22
Symptomatic cholelithiasis	42 080 (6.8)	1762 (11.6)	<.001
Other	100 164 (16.1)	2542 (16.8)	.02
**Hospitals** ^b^			
Ownership			
For profit	101 792 (16.3)	4377 (28.9)	<.001
Nonprofit	454 116 (72.9)	9607 (63.4)	
Other profit	63 653 (10.2)	1101 (7.3)	
Missing	3059 (0.5)	60 (0.4)	
Bed size, No.			
<250	266 302 (42.8)	5599 (37.0)	<.001
250 to <500	225 102 (36.2)	5576 (36.8)	
≥500	128 157 (20.6)	3910 (25.8)	
Missing	3059 (0.5)	60 (0.4)	
Geographic region			
Midwest	139 840 (22.5)	3308 (21.8)	<.001
Northeast	96 769 (15.5)	1951 (12.9)	
South	266 633 (42.8)	7138 (47.1)	
West	116 319 (18.7)	2688 (17.7)	
Missing	3059 (0.5)	60 (0.4)	
Teaching hospital	370 646 (59.8)	10 459 (69.3)	<.001
Inpatient surgical operations, No.			
Mean (SD)	4994.0 (5043.8)	5679.7 (5750.4)	<.001
Median (IQR)	3551 (1836-6390)	3871 (2148-6828)	<.001
**Surgeons**			
Surgeon volume, total No. of cases^c^			
Mean (SD)	61 (54)	60 (53)	.049
Median (IQR)	48 (23-82)	45 (23-82)	.001
**Postoperative outcome**			
Overall risk-adjusted complications, % (95% CI)	21.5 (21.2-21.8)	21.8 (21.0-22.7)	.28

^a^ Race and ethnicity were self-reported. Categories reflect data provided by Medicare.

^b^ Hospital characteristics were derived from the American Hospital Association hospital survey.

^c^ Surgeon volumes reflect total case volumes and were generated by dividing the Medicare case volumes by each hospital’s proportional Medicare payer mix derived by dividing the total number of Medicare discharges by total hospital admissions from the American Hospital Association survey.

**Figure.**
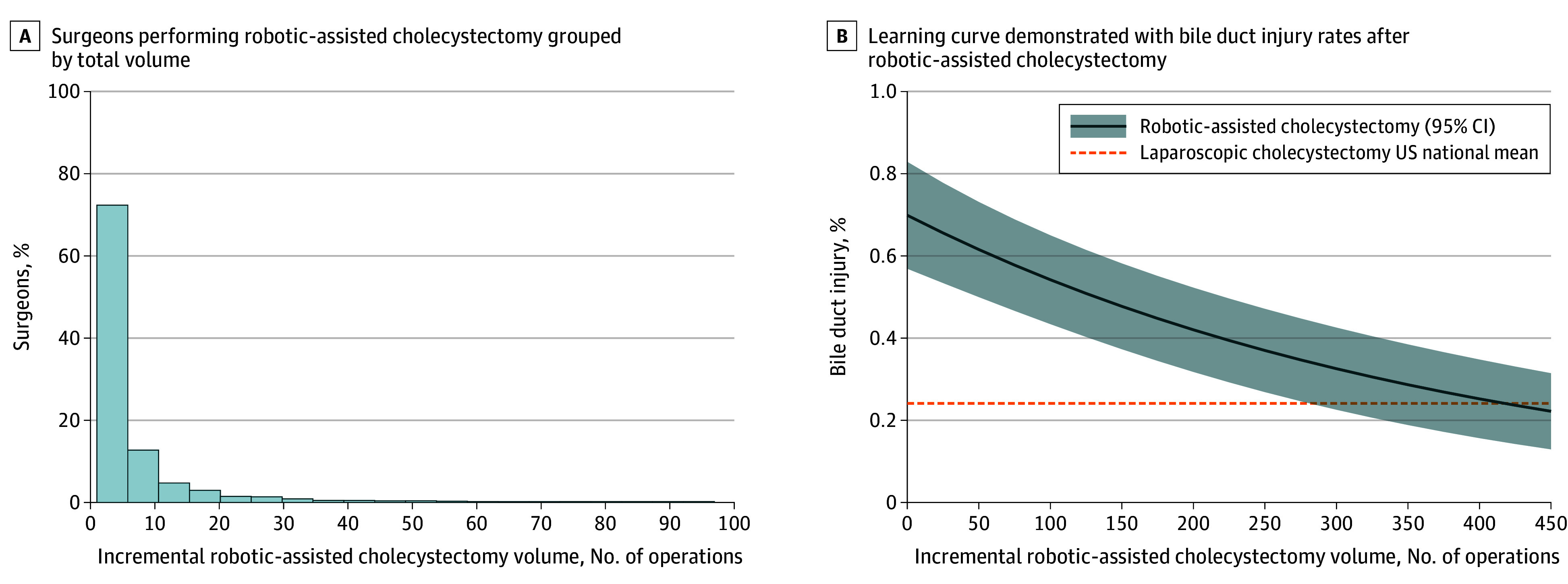
Robotic-Assisted Cholecystectomy Surgeon-Level Volume and Learning Curve A, Proportion of surgeons performing robotic-assisted cholecystectomy grouped by total volume from 2010 through 2019. B, The learning curve for robotic-assisted cholecystectomy, showing adjusted bile duct injury rates by surgeons’ incremental experience with the approach. The dashed line displays the US national mean bile duct injury rate for laparoscopic cholecystectomy derived from the Medicare cohort over the entire study period. Volumes for both x-axes reflect total volumes, not just Medicare, derived by dividing the total number of Medicare discharges by total hospital admissions from the American Hospital Association survey.

## Discussion

This study reflecting a cohort of US surgeons suggests that while increasing experience is associated with a decrease in bile duct injury rates following robotic-assisted cholecystectomy, most surgeons do not perform enough to reach equivalence with traditional laparoscopic cholecystectomy. This study may be limited by the assumption that surgeons’ first cases in Medicare claims reflect true first case(s); however, this would bias the results toward shorter learning curves as these early cases would already reflect some experience with the technology. While unmeasured confounding may also limit the conclusions (eg, extent of trainee involvement, prior cholecystostomy tube), similar patient characteristics (higher-severity diagnoses among robotic-assisted cholecystectomy, eg, cholecystitis) and similar rates of overall complications do not suggest that the robotic-assisted cohort has higher risk overall.

These data further call to question 2 contemporary practices in surgery across the United States: overall growth in robotic-assisted cholecystectomy and use of this procedure as a familiar learning case for building robotic skills or achieving local credentialing standards. Proponents of robotic-assisted cholecystectomy contend that surgeons beyond their learning curve provide safe and effective care. Even though surgical training has shifted toward greater exposure to robotics, the prolonged learning curve demonstrated in this study suggests it is worth questioning whether further uptake of robotic-assisted cholecystectomy justifies the added morbidity to reach it.

## Notes

Supplement 1. **eAppendix.** Supplemental Methods

Supplement 2. Data Sharing Statement
