# Supplementary material for: Learning Curve for Robotic-Assisted Cholecystectomy
Source: JAMA Surg. 2024 May 22;159(7):833–6. doi: 10.1001/jamasurg.2024.1221 (PMC11112490; doi:10.1001/jamasurg.2024.1221)
Supplement: Supplement 1. — eAppendix. Supplemental Methods [file jamasurg-e241221-s001.pdf]

Supplemental Online Content

Sheetz KH, Thumma JR, Kalata S, Norton EC, Dimick JB. Learning curve for robotic-assisted cholecystectomy. *JAMA Surg*. Published online May 22, 2024. doi:10.1001/jamasurg.2024.1221

eAppendix. Supplemental Methods

This supplemental material has been provided by the authors to give readers additional information about their work.

© 2024 Sheetz KH et al. *JAMA Surgery.*

eAppendix. Supplemental Methods

Overall modeling strategy:
We used multivariable logistic regression to evaluate the association between incremental robotic surgery experience at the surgeon level and bile duct injury. The model included age, race and ethnicity, sex, 29 Elixhauser comorbidities, primary diagnosis, year, hospital bed size, teaching status, and region. We specified estimates using robust standard errors.

We modeled incremental surgeon volume by identifying surgeons using discrete national provider identifiers. We grouped cases based on whether they were the surgeon's 1st through nth case, such that the bile duct injury rate at any specific volume reflects that outcome for all surgeons' nth case. For example, at 10 cases, this would estimate the bile duct injury rate for all surgeons' 10th robotic cholecystectomy in the cohort.

Sensitivity analyses:
Description of alternative modeling strategy restricted to high-volume robotic surgeons –

- Even though our analysis explicitly tests how increasing experience with robotic-assisted cholecystectomy influences bile duct injury rates, it is plausible that certain very experienced may have shorter learning curves because of global familiarity with the approach. To test this, we specified an analysis restricted to the 100 highest volume robotic-assisted cholecystectomy surgeons in the United States. The risk-adjustment specifications were identical to our main model. This analysis included 8,906 total cholecystectomies of which 3,936 (44.2%) were performed robotically. We observed a similar trend in the learning curve, with fewer cases to reach equivalence to their own average laparoscopic benchmark. However, these surgeons also had better outcomes than the national cohort for laparoscopic cases, suggesting overall greater familiarity with minimally-invasive surgery by either approach.

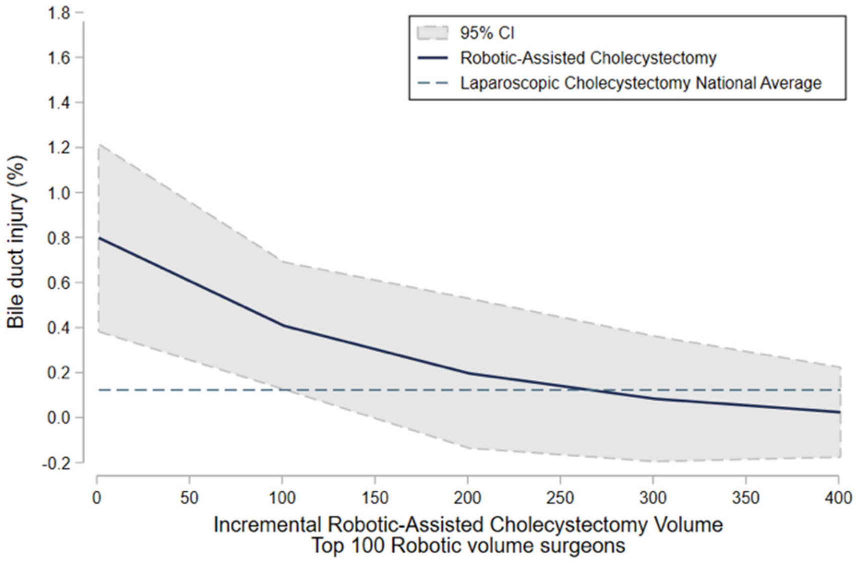

© 2024 Sheetz KH et al. *JAMA Surgery.*

Description of alternative modeling strategy using linear splines –

- Because the risk of bile duct injury may be different across the continuum of the robotic-assisted cholecystectomy learning curve, we fit another model using linear splines with knots to create four segments along the curve (<25th percentile, 25th-50th percentile, 50th-75th percentile, and >75th percentile).
- These models were similarly adjusted for patient demographics and comorbidities in the same fashion as the logistic regression model with robust standard errors.

Learning curve generated by the linear spline approach:

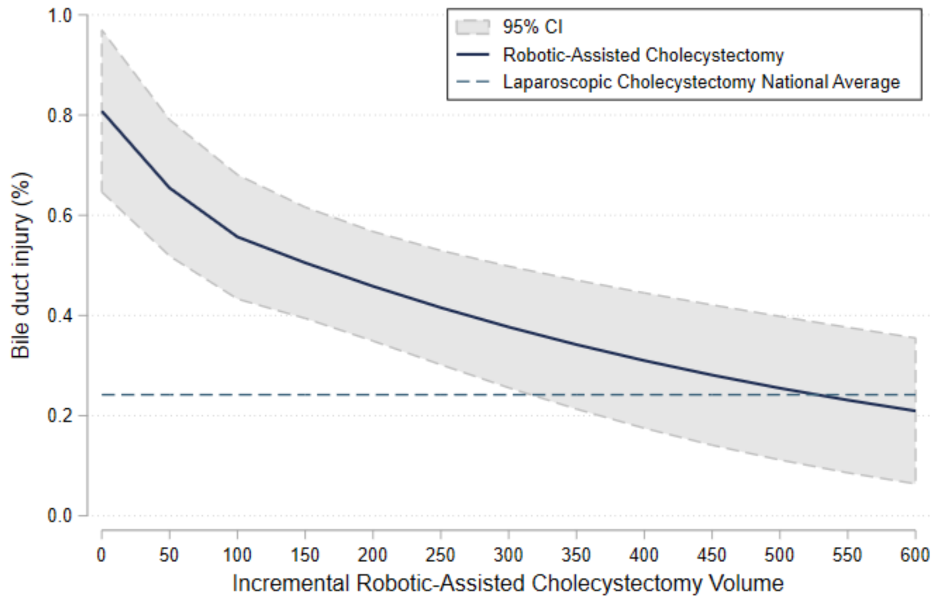

© 2024 Sheetz KH et al. *JAMA Surgery.*
