# Supplementary material for: Learning Curve for Robotic-Assisted Cholecystectomy
Source: JAMA Surg. 2024 May 22;159(7):833–6. doi: 10.1001/jamasurg.2024.1221 (PMC11112490; doi:10.1001/jamasurg.2024.1221)
Supplement: Supplement 2. — Data Sharing Statement [file jamasurg-e241221-s002.pdf]

Data Sharing Statement

Sheetz. Learning Curve for Robotic-Assisted Cholecystectomy. *JAMA Surg*. Published May 22, 2024. doi:10.1001/jamasurg.2024.1221

Data
Data available: No

Additional Information
Explanation for why data not available: Raw Medicare data is not sharable per DUA.
